# Midkine-a Is Required for Cell Cycle Progression of Müller Glia during Neuronal Regeneration in the Vertebrate Retina

**DOI:** 10.1523/JNEUROSCI.1675-19.2019

**Published:** 2020-02-05

**Authors:** Mikiko Nagashima, Travis S. D'Cruz, Antoinette E. Danku, Doneen Hesse, Christopher Sifuentes, Pamela A. Raymond, Peter F. Hitchcock

**Affiliations:** ^1^Department of Ophthalmology and Visual Sciences, and; ^2^Department of Molecular, Cellular and Developmental Biology, University of Michigan, Ann Arbor, Michigan 48105

**Keywords:** zebrafish, neurogenesis, photoreceptors, proliferation, reprogramming

## Abstract

In the retina of zebrafish, Müller glia have the ability to reprogram into stem cells capable of regenerating all classes of retinal neurons and restoring visual function. Understanding the cellular and molecular mechanisms controlling the stem cell properties of Müller glia in zebrafish may provide cues to unlock the regenerative potential in the mammalian nervous system. Midkine is a cytokine/growth factor with multiple roles in neural development, tissue repair, and disease. In *midkine-a* loss-of-function mutants of both sexes, Müller glia initiate the appropriate reprogramming response to photoreceptor death by increasing expression of stem cell-associated genes, and entering the G_1_ phase of the cell cycle. However, transition from G_1_ to S phase is blocked in the absence of Midkine-a, resulting in significantly reduced proliferation and selective failure to regenerate cone photoreceptors. Failing to progress through the cell cycle, Müller glia undergo reactive gliosis, a pathological hallmark in the injured CNS of mammals. Finally, we determined that the Midkine-a receptor, anaplastic lymphoma kinase, is upstream of the HLH regulatory protein, Id2a, and of the retinoblastoma gene, *p130*, which regulates progression through the cell cycle. These results demonstrate that Midkine-a functions as a core component of the mechanisms that regulate proliferation of stem cells in the injured CNS.

**SIGNIFICANCE STATEMENT** The death of retinal neurons and photoreceptors is a leading cause of vision loss. Regenerating retinal neurons is a therapeutic goal. Zebrafish can regenerate retinal neurons from intrinsic stem cells, Müller glia, and are a powerful model to understand how stem cells might be used therapeutically. Midkine-a, an injury-induced growth factor/cytokine that is expressed by Müller glia following neuronal death, is required for Müller glia to progress through the cell cycle. The absence of Midkine-a suspends proliferation and neuronal regeneration. With cell cycle progression stalled, Müller glia undergo reactive gliosis, a pathological hallmark of the mammalian retina. This work provides a unique insight into mechanisms that control the cell cycle during neuronal regeneration.

## Abstract (precis)

In the retina of zebrafish, Müller glia have the ability to reprogram into stem cells capable of regenerating all classes of retinal neurons and restoring visual function. Understanding the cellular and molecular mechanisms controlling the stem cell properties of Müller glia in zebrafish may provide cues to unlock the regenerative potential in the mammalian nervous system. Midkine is a cytokine/growth factor with multiple roles in neural development, tissue repair, and disease.

## Introduction

Cell division is an essential biological process during development, homeostasis, and repair. In the CNS of adult mammals, stem cells reside in specialized niches, and these cells maintain the ability to divide and generate new neurons (Kriegstein and Alvarez-Buylla, 2009; Ming and Song, 2011). In the vertebrate retina, Müller glia harbor molecular features of stem and progenitor cells (Dyer and Cepko, 2000). In mammals, Müller glia respond to injury by partial dedifferentiation and entering the G_1_ phase of the cell cycle (Bringmann et al., 2006). However, in general, this reprogramming does not lead to cell division, and structural remodeling and the loss of retinal homeostasis are the typical sequalae (Bringmann et al., 2009; Karl and Reh, 2010; Hamon et al., 2016). Importantly, in the limited instances where regeneration does occur, new neurons functionally integrate into existing synaptic circuits (Jorstad et al., 2017; Yao et al., 2018), indicating that in the mammalian retina the limitations of neuronal regeneration hinge on a more complete neurogenic response in Müller glia.

In zebrafish, Müller glia can adopt the features of stem cells (Karl and Reh, 2010; Goldman, 2014; Gorsuch and Hyde, 2014; Lenkowski and Raymond, 2014; Hamon et al., 2016). In uninjured retinas, Müller glia reside in a quiescent state and function to maintain retinal homeostasis. Neuronal death triggers Müller glia to reprogram into a stem cell-like state, enter the cell cycle, and undergo a single asymmetric division to produce rapidly dividing, multipotent retinal progenitors with the ability to regenerate retinal neurons (Nagashima et al., 2013; Lenkowski and Raymond, 2014). Several signaling pathways have been identified that regulate the initial response of Müller glia (Karl and Reh, 2010; Goldman, 2014; Gorsuch and Hyde, 2014; Lenkowski and Raymond, 2014; Hamon et al., 2016). Ascl1, Lin28, and Stat3 have been identified as “core” transcriptional regulators that govern signaling cascades required for Müller glia to divide (Fausett and Goldman, 2006; Ramachandran et al., 2010; Nelson et al., 2012).

Midkine is a growth factor/cytokine that has multiple roles in neural development, repair, and disease (Sakamoto and Kadomatsu, 2012; Winkler and Yao, 2014; Sorrelle et al., 2017). In malignant tumors, Midkine promotes proliferation and metastasis (Muramatsu, 2011) and is also involved in CNS inflammation (Muramatsu, 2011; Weckbach et al., 2011; Herradon et al., 2019). The diverse functions of Midkine are transduced through receptors, which may function individually or as members of a multiprotein complex (Muramatsu, 2011; Weckbach et al., 2011; Xu et al., 2014). During retinal development in zebrafish, *midkine-a* is expressed by retinal progenitors and functions to govern elements of the cell cycle (Calinescu et al., 2009b; Uribe and Gross, 2010; Luo et al., 2012). Postmitotic neurons downregulate *midkine-a*. Retinal injury rapidly induces *midkine-a* in Müller glia (Calinescu et al., 2009b; Gramage et al., 2014, 2015). Induction of *midkine-a* following injury has been reported for a variety of tissues with the capacity to regenerate (Ochiai et al., 2004; Lien et al., 2006), suggesting that Midkine may universally regulate aspects of tissue regeneration. The molecular mechanisms whereby Midkine governs regeneration are not well understood.

Using a Midkine-a loss-of-function mutant, we demonstrate that, following a retinal injury, Midkine-a is required for reprogrammed Müller glia to progress from G_1_ to S phases of the cell cycle. Following photoreceptor death, Müller glia in Midkine-a mutants reprogram into a stem cell state and enter G_1_ phase of the cell cycle. However, for the vast majority of Müller glia, subsequent entry into the S phase and mitotic division are blocked, resulting in failure to regenerate cone photoreceptors. Further, Midkine-a is required for the upregulation of *id2a*, which inhibits the retinoblastoma (Rb) family of cell cycle inhibitors. In addition, the G_1_-arrested Müller glia undergo reactive gliotic remodeling, hallmark of pathology in the mammalian retina. Finally, we provide evidence that activation of the Midkine receptor, anaplastic lymphoma kinase (Alk), is required for proliferation in Müller glia.

## Materials and Methods

### 

#### 

##### Zebrafish.

Fish were maintained at 28°C on a 14/10 h light/dark cycle with standard husbandry procedures. Adult WT, AB-strain zebrafish (*Danio rerio*; ZIRC, University of Oregon, Eugene, OR) and the transgenic reporter line *Tg(gfap:eGFP)^mi2002^* (Bernardos and Raymond, 2006) were of either sex and used between 6 and 12 months of age. All animal procedures were approved by the Institutional Animal Care and Use Committee at the University of Michigan.

##### CRISPR-Cas9-mediated targeted mutation of midkine-a.

Targeted mutations in the *midkine-a* locus were introduced using CRISPR-Cas9 (Hwang et al., 2013). Briefly, ZiFit software (http://zifit.partners.org/ZiFiT/) was used to identify guide RNA target sequence for *midkine-a*. Oligos to the target sequencing (GGC AGC TGC GTG GCC AAT AAC GG) were annealed and subcloned into the pT7 gRNA vector (Addgene plasmid # 46759; http://n2t.net/Addgene:46759; RRID:Addgene_46759; Nasevicius and Ekker, 2000; Hwang et al., 2013; Jao et al., 2013). The subcloned vector was digested with BamHI, and sgRNA was transcribed with MEGAshortscript T7 kit (Thermo Fisher Scientific). To synthesize *cas9* mRNA, pCS2-nCas9n plasmid (Addgene plasmid # 47929; http://n2t.net/addgene:47929; RRID:Addgene_47929) and mMessage mMachine SP6 *in vitro* transcription kits (Thermo Fisher Scientific) were used. Purification of sgRNA and mRNA was performed using mirVana miRNA isolation kit (Thermo Fisher Scientific) and RNeasy Mini Kit (QIAGEN). Single-cell stage embryos were injected with 1 nl solution, containing 150 pg *cas9* mRNA and 100 pg sgRNA diluted in 1× Danieux buffer with 2.5% phenol red. F0 embryos were raised to adulthood and then outcrossed with AB-WT animals. To screen potential mutants in F1 generation, genomic DNA fragment containing the *midkine-a* target site was amplified with primers (forward: TGACTTTGAAGCTTATTGACGCTG; reverse: GTGCAGGGTTTGGTCACAGA) and was subjected to T7 endonuclease assay. PCR products with potential indel mutation in the *midkine-a* gene were sequenced and analyzed with National Center for Biotechnology Information Basic Local Alignment Search Tool and ExPaSy translate tool (www.expasy.org). F1 progenies with indel mutation were in-crossed, and homozygous F2 mutants were identified.

##### Western blots.

Western blot analyses were performed as previously described (Calinescu et al., 2009a). Briefly, proteins were extracted from the heads of 30–50 WT and *mdka^mi5001^* embryos or adult retinas (6 retinas from 3 animals per sample) in cold RIPA lysis buffer containing protease and phosphatase inhibitor mixture (Cell Signaling Technology). Proteins were separated in 12% Mini-PROTEIN TGX Precast gel (Bio-Rad) and were transferred to PVDF membranes (GenHunter). After blocking in 5% nonfat dry milk in Tris-buffered saline containing 0.3% Tween 20, membranes were incubated with rabbit anti-Midkine-a antisera or rabbit anti-STAT3 (Nelson et al., 2012) followed by HRP-conjugated secondary antibody (1:1000) (Calinescu et al., 2009a). Immunolabeled proteins were detected using the enhanced ECL detection system for chemiluminescence assay (GE Healthcare). Actin was used as a loading control.

##### RNAseq.

Embryos at 30 hpf were manually dechlorinated. Deyolking was performed by triturating with glass pipette in cold Ringer's solution containing 1 mm EDTA and 0.3 mm PMSF in isopropanol. Total RNA from 30 embryos was extracted using TRIzol (Invitrogen). Purity of RNA was analyzed with Bioanalyzer (Agilent Technologies). Samples with an RNA integrity number of acceptable quality (>7) were used for Illumina RNA-seq library preparation. Deep sequencing was performed on an Illumina GAIIx Sequencer (Illumina).

##### Read quality trimming and quality assessments.

Trim Galore! (version 0.2.7; Babraham Institute) was used to trim adapter sequences and poor-quality bases (below Phred of 20) from the reads while removing any reads that were <20 nt long, using the default parameters. Trim Galore! makes use of cutadapt (version 1.4.2) (-f fastq-e 0.1-q 20-O 1-a AGATCGGAAGAGC file.fq.gz). The quality of the reads was assessed before and after trimming with FastQC (version 0.10.1).

##### Read mapping and gene-level quantitation.

Quality trimmed and filtered reads were aligned to release 83 of the GRCz10 Ensembl genome build with bowtie2 (version 2.2.6), and gene-level quantitation was performed with RSEM (version 1.2.22). This was done using the rsem-calculate-expression command from RSEM, which calls bowtie2 (-sensitive -dpad 0 -gbar 99999999 -mp 1,1 -np 1 score-min L,0,–0.1) and streams reads into RSEM for quantitation.

##### Differential expression analysis and annotation.

The gene-level counts output from RSEM were filtered to remove noise before normalization with trimmed means of M, such that only genes with a FPKM (fragments per kilobase of exon per million reads mapped) value > 1 in all replicates of any genotype were retained. Counts per million were determined using edgeR (version 3.10.2), genes with a counts per million < 1 in all samples were removed, and remaining counts were trimmed means of M normalized. Limma (version 3.24.15) was used to voom transform the filtered count data by empirically deriving and applying quality weights to the samples. These weighted values were used to calculate differential expression using limma. Annotations for each gene were added using biomaRt (version 2.24.0), including both the *D. rerio* Entrez gene identifiers and the corresponding *Mus musculus* Entrez orthologous gene identifiers.

##### Gene ontology and pathway analysis overview of workflow.

Gene ontology term enrichment analysis was performed using a log2-fold change (log2FC) ranked list from limma (log2FC > 1 and false discovery rate < 0.05) as input into clusterProfiler (version 2.2.4). This analysis determines which Molecular Function, Biological Process, or Cellular Component gene ontology terms are positively or negatively enriched in the mutant embryos compared with WT, at a false discovery rate ≤ 0.05, while taking into account the magnitude and direction of change. Pathway database, Reactome pathway analyses, were used. A log2FC ranked list of all differential gene expression data from limma (log2FC > 1 and false discovery rate < 0.05) was input into ReactomePA (1.12.3).

All the zebrafish genes in the dataset were manually annotated with their murine orthologs using biomaRt, and a Reactome pathway analyses was performed using zebrafish gene annotations (from zebrafish differential expression data) and zebrafish pathway annotations. The analysis was performed with zebrafish Entrez gene identifiers to determine the Reactome pathways that were positively or negatively enriched at a false discovery rate ≤ 0.05.

##### EdU and BrdU labeling.

Proliferating cells were labeled with the S-phase markers, EdU (Thermo Fisher Scientific) or BrdU (Millipore Sigma). Embryos were incubated on ice in 1.5 mm EdU dissolved in embryo rearing solution containing 15% diethylsulfoxide for 20 min. Embryos were then returned to room temperature for 10 min before fixation. EdU-labeled cells were visualized using Click-iT Assay kit (Thermo Fisher Scientific). To label dividing cells in adults, animals were housed in the fish system water containing 5 mm BrdU.

##### Light lesion.

To selectively damage photoreceptors, an ultra-high intensity light lesion was used as previously described (Bernardos et al., 2007). In brief, zebrafish were exposed to 120,000 lux light from an EXFO X-Cite 120 W metal halide lamp for 30 min and then returned to the aquarium system.

##### Histology.

Embryos and eye balls were fixed in 4% PFA with 5% sucrose in PB, pH 7.4, at room temperature for 2 h or 4°C overnight, respectively. After rinsing with 5% sucrose in PBS, tissues were cryoprotected, embedded, and sectioned at 6 μm. Immunocytochemistry was performed as previously described (Bernardos et al., 2007). Briefly, sections were blocked with blocking reagent containing 20% normal goat serum/0.5% Triton X-100 in PBS, pH 7.4, with 0.1% sodium azide. Primary antibodies (mouse anti-PCNA, Millipore Sigma P8825; mouse anti-Zpr1, Zebrafish International Resource Center; mouse anti-Zpr3, Zebrafish International Resource Center; mouse anti-Zrf1, Zebrafish International Resource Center; mouse anti-zonula occludens 1 [ZO1], Thermo Fisher Scientific, ZO1–1A12; guinea pig anti-Rx1 (Nagashima et al., 2013); mouse anti-Sox2, Genetex, T+GTX124477; mouse anti-BrdU, Abcam, ab6326; mouse anti-pStat3, MBL, D128-3; rabbit anti-phosphorylated anaplastic lymphoma kinase [pAlk], Abcam, ab111865: chick anti-GFP, Abcam, ab13970) diluted with diluting reagent containing 1% normal goat serum/0.5% Triton X-100 in PBS, pH 7.4, with 0.1% sodium azide were incubated 4°C overnight. AlexaFluor secondary antibody (Thermo Fisher Scientific) incubation was performed at room temperature for 2 h. Slides were stained with Hoechst 33342 (Thermo Fisher Scientific) for nuclear staining. For BrdU detection, sections were pretreated with 2N HCl in PBS and 0.5% Triton X-100 in PBS for 30 min before blocking.

##### Flat-mount retinal immunocytochemistry.

Retinas were isolated from the eyes of dark-adapted zebrafish and fixed overnight at 4°C in 4% PFA in PB with 5% sucrose, pH 7.4. Immunocytochemistry was performed as previously described (Nagashima et al., 2017). Briefly, retinas were treated with 10 mm sodium citrate in 0.05% Tween 20, pH 6.0, in boiling water for 5 min. Free-floating retinas were blocked with 10% normal goat serum/1% Tween 20/1% Triton X-100/1% DMSO in PBS, pH 7.4, with 0.1% sodium azide for 2 h. Primary and secondary antibodies were diluted in 0.5% normal goat serum/1% Tween 20/1% Triton X-100/1% DMSO in PBS, pH 7.4, with 0.1% sodium azide, and incubations were performed at room temperature overnight.

##### Microscopy and image analysis.

Retinal cross sections and flat-mount retinas were imaged with DM6000 Upright Microscope System and TCS SP5 confocal microscope (Leica Microsystems), respectively. Adobe Photoshop CS6 Extended (Adobe Systems), Application Suite X (Leica Microsystems), ImageJ (https://imagej.nih.gov/ij/), or Imaris 7.6.1 (Bitplane) were used for image analysis, 3D reconstruction, and movie production.

##### qPCR.

Total RNA from whole retinas was extracted using TRIzol (6 retinas from 3 fish per sample) (Invitrogen). RNA was quantified using Nanodrop spectrophotometer (Thermo Fisher Scientific). Reverse transcription and qPCR were performed according to the manufacturer's instructions using QIAGEN QuantiTec Reverse Transcription kit and Bio-Rad IQ SYBR Green Supermix, respectively. Reactions were performed using a CFX384 Touch Real-Time PCR Detection Systems (Bio-Rad). Primer sequences are listed in the supporting Table 1.

**Table 1.** qPCR primer sequences

Primer name	Sequence
gpia forward	TCC AAG GAA ACA AGC CAA GC
gpia reverse	TTC CAC ATC ACA CCC TGC AC
ascl1a forward	GGG CTC ATA CGA CCC TCT GA
ascl1a reverse	TCC CAA GCG AGT GCT GAT ATT T
stat3 forward	GAG GAG GCG TTT GGC AAA
stat3 reverse	TGT GTC AGG GAA CTC AGT GTC TG
lin28 forward	CGT GCG GAT GGG CTT CGG ATT TC
lin28 reverse	GGC CCC GTC ACT TGT AAG GAC TC
ccnd1 forward	CTT ACA CAG AGA AGT TGT G
ccnd1 reverse	GGC AAG GAA GTG TTC AAT G
ccne1 forward	ACC GGA GAC CTC TGA CTG CT
ccne1 reverse	AGC AGG CAG CTC AGC CCT TA
ccna2 forward	CGC TAA ACA GGG GTC TGG GC
ccna2 reverse	GGT GCT TTC TTG GAG CAC GC
cdk4 forward	GCG TTC GGG TGC AGA CCA AT
cdk4 reverse	TGA TCC GTC CTG AGG GTC GC
cdk6 forward	TCT CAC CGT GTG GTT CAT CGG
cdk6 reverse	ATG TCA CAA CCA CCA CGG AAG T
id2a forward	CAG ATC GCG CTC GAC TCC AA
id2a reverse	CAG GGG TGT TCT GGA TGT CCC
p130 forward	AGT CGA GTA ACC GAG CCT GGA
p130 reverse	TGG GCA CTG ATG AGC GAC AC

##### ALK inhibitor treatment.

Zebrafish were housed from 24 to 72 h post lesion (hpl) in system water containing 10 μm TAE684 (Abcam, ab142082) constructed from a 20 mm stock solution in 0.1% DMSO. Control groups were housed in system water containing the 0.1% DMSO. Solutions were changed daily.

##### Experimental design and statistical analysis.

In radial sections, cells counted in three nonadjacent sections in each retina were averaged. Three to seven retinas were analyzed. In flat-mount preparation, ZO1 profiles with perimeter >3.5 μm were identified as cone photoreceptors. For each retina, cones in 5625 μm^2^ area were counted using National Institutes of Health ImageJ (https://imagej.nih.gov/ij/). A total of six retinas were analyzed.

For qPCR experiment, three biological replicates were prepared for each time point, and three technical replicates were evaluated for each sample. For quantification of fold changes, ΔΔ C(t) method was used, and the housekeeping gene, *gpia*, was used to normalize the data (Livak and Schmittgen, 2001).

Statistical analysis was performed in JMP pro software using the nonparametric Mann-Whitney-Wilcoxon and ANOVA with *post hoc* Tukey HSD test (SAS Institute). A *p* value ≤ 0.03 was considered significant.

## Results

### Loss-of function mutant, *mdka^mi5001^*

**Figure 1.**
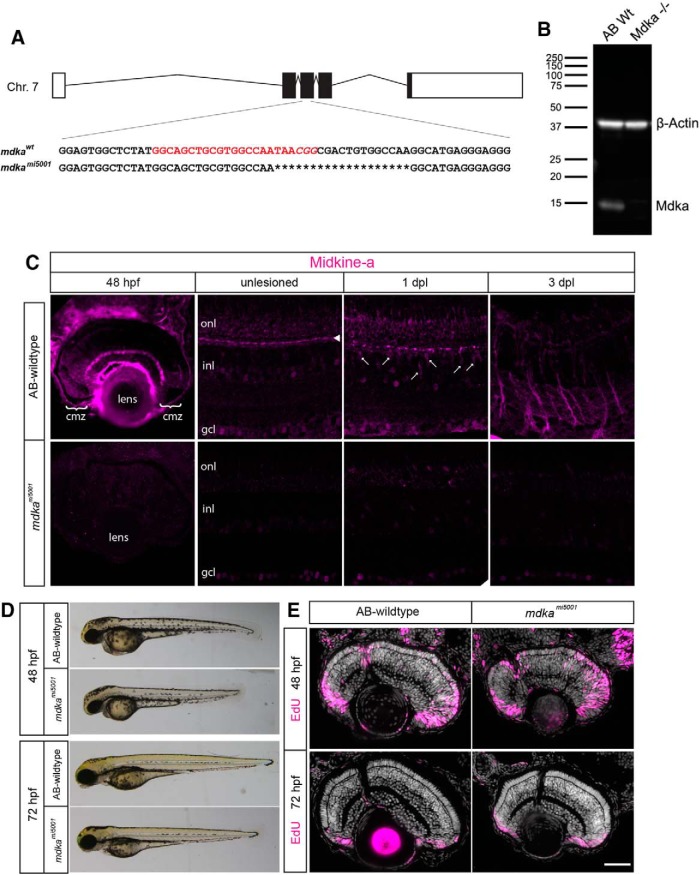
Midkine-a governs cell cycle kinetics during retinal development. ***A***, Schematic representation of the *midkine-a* locus on chromosome 7. The gene consists of five exons. Red represents the gRNA target sequence located at exon 3. ***B***, Using larvae at 48 h post fertilization (hpf), Western blot analysis for Midkine-a confirms lack of protein in the *mdka^mi5001^* mutant. ***C***, Immunocytochemistry for Midkine-a in embryonic (48 hpf), adult, and regenerating retinas. In unlesioned retina, Midkine-a immunoreactivity is detected in horizontal cells (arrowhead) (Gramage et al., 2015). Photoreceptor cell death induces Midkine-a in reprogrammed Müller glia (arrows) at 1 dpl. Midkine-a distributes radial processes of Müller glia at 3 dpl. ***D***, Lateral views of larvae at 48 and 72 hpf: AB-WT and *mdka^mi5001^* mutant. ***E***, Proliferation assay with EdU labeling at 48 hpf. Compared with WT, there is an increased number of EdU-labeled cells in the retinas of *mdka^mi5001^* mutants. Retinal lamination and cellular differentiation are delayed, but not blocked in the *mdka^mi5001^* at 72 hpf. cmz, Ciliary marginal zone; onl, outer nuclear layer; inl, inner nuclear layer; gcl, ganglion cell layer. Scale bar, 40 μm. (see extended data, Table 1-1, and Table 1-2).

We generated a CRISPR-Cas9-mediated Midkine-a loss-of-function mutant, *mdka^mi5001^*, which carries a 19 bp deletion in the exon three of *midkine-a*. This deletion results in a predicted premature stop codon (Fig. 1*A*) and absence of protein in Western blot analysis (Fig. 1*B*). Immunostaining of both larval and adult retinas showed absence of protein in these tissues (Fig. 1*C*). *Mdka^mi5001^* larvae progress normally through early developmental stages and at 48 hpf show only slight reduction in body pigmentation, shortened body length, and smaller eyes (Fig. 1*D*). The pigmentation defect recovers by 72 hpf (Fig. 1*D*). Notably, the *mdka^mi5001^* mutants replicate the delayed retinal development described previously following morpholino-mediated knockdown of Midkine-a (Fig. 1*E*) (Luo et al., 2012).

10.1523/JNEUROSCI.1675-19.2019.t1-1 Table 1-1 Differential gene expression analysis of whole embryos at 30 hours post fertilization. Download Table 1-1, XLSX file

10.1523/JNEUROSCI.1675-19.2019.t1-2 Table 1-2 Pathway analysis of whole embryos at 30 hours post fertilization. Download Table 1-2, XLSX file

### Transcriptome analysis

Larvae from adult *mdka^mi5001^* mutants were initially evaluated using transcriptome analysis of whole embryos at 30 hpf. This identified 638 differentially expressed genes (log2 fold change ≥ 1 and a false discovery rate ≤ 0.05) (Table 1-1). Pathway-level analysis with the Reactome tool identified 181 pathways that were differentially regulated (156 upregulated and 25 downregulated; Table 1-2). Of the 156 upregulated pathways, 33 were related to cell cycle regulation (Table 1-2). On the other hand, pathways related to maturation of the nervous systems are downregulated (Table 1-2). These data validated our previous study (Luo et al., 2012) and directed us to evaluate the role of Midkine-a in regulating proliferation in Müller glia.

### Regeneration of cone photoreceptors is compromised in the *mdka^mi5001^* mutant

Persistent, growth-associated neurogenesis is a hallmark of teleost fish (Hitchcock et al., 2004). In the growing eye and retina, stem and progenitor cells at the ciliary marginal zone generate new retinal neurons, with the exception of rod photoreceptors (Cerveny et al., 2012). Fate-restricted, proliferating rod precursors, derived from sporadic division of Müller glia and sequestered in the outer nuclear layer, selectively give rise new rod photoreceptors (Raymond and Rivlin, 1987; Bernardos et al., 2007; Stenkamp, 2011). This growth-associated neurogenesis occurs normally in the retinas of *mdka^mi5001^* mutants, and there is no apparent alteration in the maturation or variety of cell types in the *mdka^mi5001^* retina (Fig. 1*E*).

**Figure 2.**
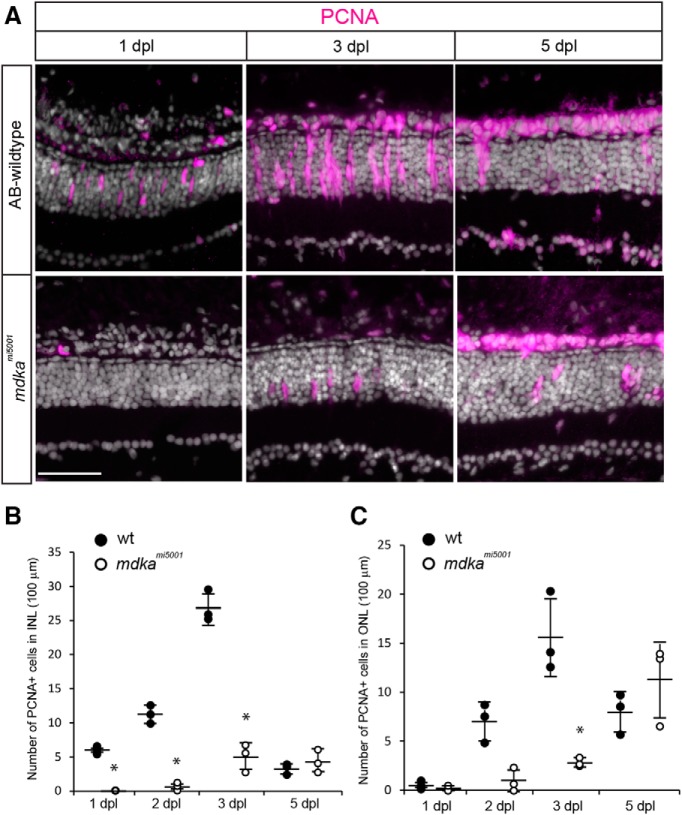
In the *mdka^mi5001^* mutant, Müller glia fail to proliferate in response to photoreceptor cell death. ***A***, Immunocytochemistry for PCNA (magenta) in WT and *mdka^mi5001^* at 1 and 3 dpl. In WT, Müller glia become positive for PCNA at 1 dpl. At 3 dpl, PCNA^+^ progenitors form neurogenic clusters. Mutant retinas lack PCNA-labeled cells at 1 dpl. Very few, isolated cells are positive for PCNA in the *mdka^mi5001^* at 3 dpl. ***B***, ***C***, The number of PCNA^+^ cells in WT and *mdka^mi5001^* in the inner (***B***; 1 dpl: *p* = 0.0017; 2 dpl: *p* < 0.0001; 3 dpl: *p* < 0.0004, ANOVA with post hoc Tukey; F ratio = 116.1834) and outer (***C***; 3 dpl; *p* = 0.0001, ANOVA with post hoc Tukey; F ratio = 17.5589) nuclear layers. The *mdka^mi5001^* retinas have significantly less PCNA^+^ cells at 1, 2, and 3 dpl compared with WT retinas. A total of 3 sections were counted and averaged in each retina. A total of 3 retinas were analyzed. Scale bar, 30 μm. *p < 0.01.

In response to neuronal cell death, Müller glia in zebrafish dedifferentiate and undergo a single asymmetric division to produce retinal progenitors, which rapidly divide, migrate to areas of cell loss, and differentiate to replace the ablated neurons (Nagashima et al., 2013). To assess photoreceptor regeneration in the *mdka^mi5001^*, we used a photolytic lesion that selectively kills photoreceptors; photoreceptors undergo apoptotic cell death by 1 day post lesion (dpl) (Vihtelic and Hyde, 2000; Bernardos et al., 2007). In WT retinas, by 1 dpl, Müller glia can be labeled with antibodies against the late G_1_ or early S phase marker, PCNA and, by 3 dpl, the Müller glia-derived progenitors form radial neurogenic clusters that span the inner nuclear layer (Fig. 2*A*). In contrast, in the *mdka^mi5001^* retinas, PCNA labeling was completely absent at 1 dpl, and only a few cells were PCNA^+^ at 3 dpl (Fig. 2*A*,*B*). Based on the size and location of their nuclei, we infer that these cells are Müller glia. However, at 5 dpl, there were no differences in the number of PCNA^+^ cells in the outer nuclear layer of WT and mutant retinas (Fig. 2*A*,*C*). In WT retinas, photoreceptor progenitors in the outer nuclear layer begin withdrawing from the cell cycle at 4 dpl (Bernardos et al., 2007). This, coupled with the delayed proliferation in the mutant retinas, can explain the similarity in the number of PCNA^+^ cells in the outer nuclear layer at 5 dpl.

**Figure 3.**
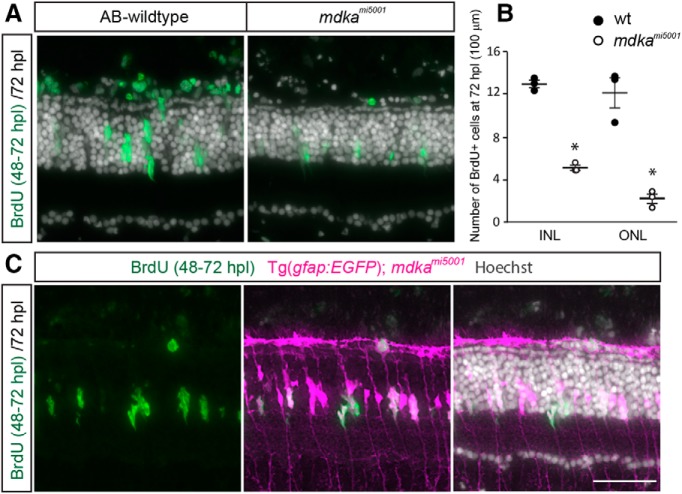
Some Müller glia in the *mdka^mi5001^* progress through the cell cycle. ***A***, ***B***, BrdU labeling between 48 and 72 hpl in WT and *mdka^mi5001^* mutant retinas. In WT, Müller glia-derived progenitors are labeled with BrdU. In *mdka^mi5001^* mutant at 72 hpl, fewer cells in the inner nuclear layer (INL) and outer nuclear layer (ONL) are labeled with BrdU compared with WT (INL: *p* < 0.0001; ONL: *p* = 0.0008, ANOVA with post hoc Tukey). ***C***, BrdU labeling (green) in transgenic reporter line, *Tg(gfap:eGFP)^mi2002^*, in the *mdka^mi5001^* mutant background. *Tg(gfap:eGFP)^mi2002^* is pseudocolored in magenta. Scale bar, 30 μm. *p < 0.01.

We next asked whether PCNA^+^ cells in *mdka^mi5001^* are capable of progressing further through the cell cycle. We exposed WT and mutants to BrdU between 48 and 72 hpl and killed the animals immediately for histology. In WT retinas at 3 dpl, BrdU^+^ cells were present in the inner and outer nuclear layers, and we infer that these BrdU^+^ cells are Müller glia-derived progenitors (see Nagashima et al., 2013). In the *mdka^mi5001^* mutants at 3 dpl, BrdU^+^ cells were also observed in the inner and outer nuclear layers, though, relative to WT retinas, there were significantly fewer cells in the mutant retinas (Fig. 3*A*,*B*; compare Fig. 2*A*). To establish the identity of the BrdU^+^ cells in the inner nuclear layer in the mutant retinas, the transgenic reporter line, *Tg(gfap:eGFP)^mi2002^* (Bernardos and Raymond, 2006), was crossed into the *mdka^mi5001^* background. This showed that, in 3 dpl, the BrdU^+^ cells in the inner nuclear layer are Müller glia. The presence of a few closely paired nuclei indicates that some of these Müller glia can progress through the cell cycle (Fig. 3*C*). These results show, in the *mdka^mi5001^* mutants, a few Müller glia can progress through the cell cycle, but relative to WT animals, many fewer of these cells divide and their division is significantly delayed.

**Figure 4.**
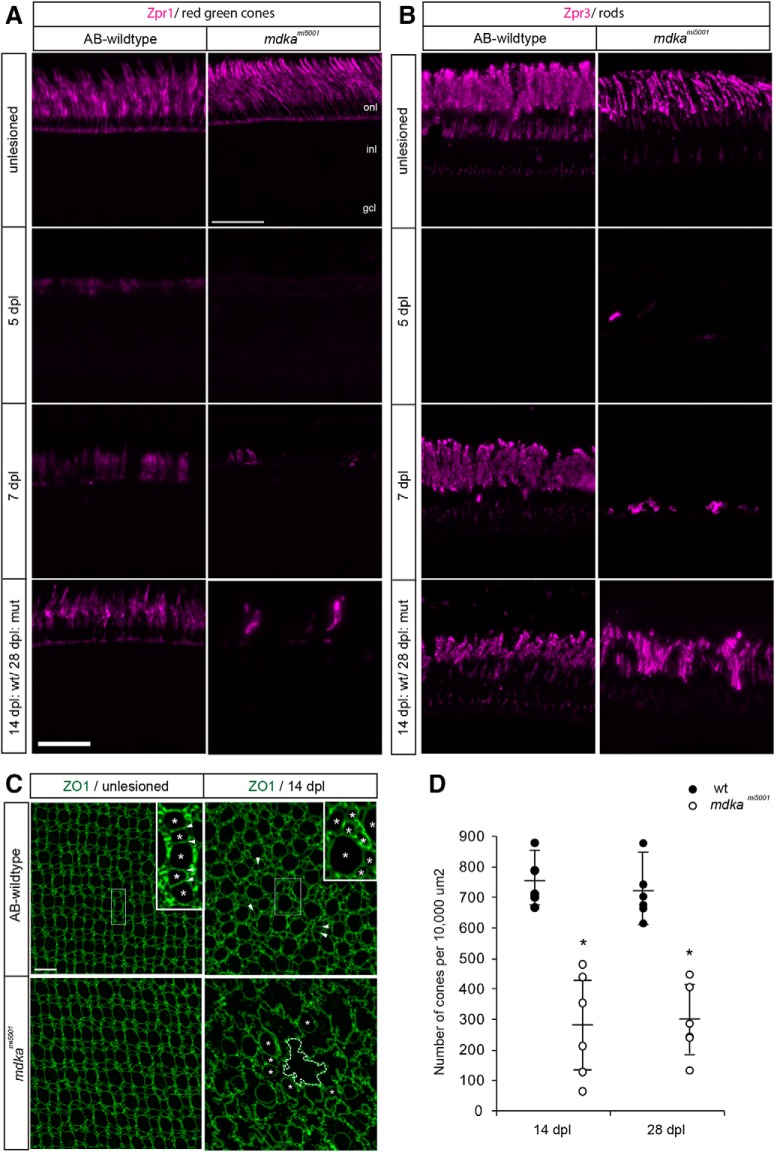
The *mdka^mi5001^* mutant retinas fail to regenerate cone photoreceptors. ***A***, Immunocytochemistry for red/green cone photoreceptor marker, Zpr1. In WT retina, immature cone photoreceptors start to appear at 5 dpl, and regeneration largely completes by 14 dpl. In the *mdka^mi5001^* mutant, regenerating photoreceptors are absent at 5 dpl. At 7 dpl, very few cone photoreceptors appear. The number of cone photoreceptors is less at 14 dpl compared with WT. ***B***, Immunocytochemistry for rod photoreceptor marker Zpr3 following lesion. In WT retina, regenerating rod photoreceptors appear by 7 dpl. In the *mdka^mi5001^* retinas, rod photoreceptors slowly regenerate by 28 dpl. ***C***, Flat-mounted retinal preparation immunostained with ZO1 in unlesioned and 14 dpl. In unlesioned retina of both WT and *mdka^mi5001^*, cone photoreceptors form a crystalline mosaic array in the planar apical surface of the retina (Livak and Schmittgen, 2001; Nagashima et al., 2017). Higher magnification of boxed region indicates the alignment of cones in the mosaic array (asterisks) with flattened cell boundaries (arrowheads). At 14 dpl in WT, cone photoreceptors regenerate (asterisks), although the crystalline mosaic array is not restored. In the *mdka^mi5001^* retina, cone profiles are instead replaced by irregularly shaped, expanded Müller glial apical processes (dotted line). ***D***, Counts of ZO1-labeled cone photoreceptors at 14 and 28 dpl. Significantly fewer cones are regenerated in the *mdka^mi5005^* mutant (white) compared with WT (gray). *n* = 6. 14 dpl: *p* = 0.0051; 28 dpl: *p* = 0.0051, nonparametric Mann-Whitney-Wilcoxon. onl, Outer nuclear layer; inl, inner nuclear layer; gcl, ganglion cell layer. Scale bars: ***A***, ***B***, 30 μm; ***C***, 10 μm. *p < 0.01.

We next assayed photoreceptor regeneration using specific cone and rod photoreceptor markers, Zpr1 and Zpr3, respectively, and flat-mount retinal preparations immunostained with the cell junction marker, ZO1. In WT retinas, regenerated cones appear as early as 5 dpl, and by 14 dpl the regeneration of cone photoreceptors is largely complete (Fig. 4*A*,*C*,*D*). In contrast, at 5 dpl in the *mdka^mi5001^* retinas, regenerated cones are nearly completely absent, and at 7 dpl only a few immature cone photoreceptors are present (Fig. 4*A*). The absence of regenerated cones persists through 14 and 28 dpl, demonstrating that, in the mutant retinas, regeneration of cone photoreceptors is not simply delayed (Fig. 4*D*). These results indicate that cone photoreceptor regeneration is permanently compromised in the absence of Midkine-a. In contrast, rod photoreceptors are regenerated slowly but to apparently normal numbers in the *mdka^mi5001^* mutants (Fig. 4*B*). In WT retinas, mature rod photoreceptors appear between 7 and 14 dpl (Fig. 4*B*), whereas in the *mdka^mi5001^* mutants, rod photoreceptors are immature at 7 dpl but completely replenished by 28 dpl (Fig. 4*B*). Together, these results demonstrate that Midkine-a is required for Müller glial to proliferate in response to cell death, and the absence of Midkine-a leads to a failure of cone photoreceptor regeneration. In contrast, rod photoreceptors regenerate normally in the mutants.

### Müller glia in the *mdka^mi5001^* mutants undergo gliotic remodeling

**Figure 5.**
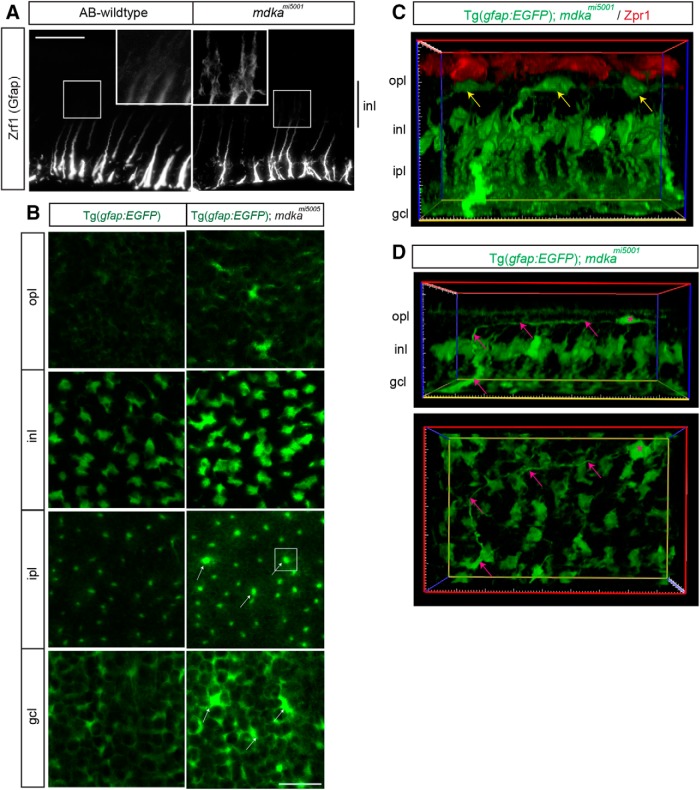
Following photoreceptor death, Müller glia in the *mdka^mi5001^* mutant undergo gliotic remodeling. ***A***, Immunocytochemistry for Gfap in WT and *mdka^mi5001^* retinas at 28 dpl. In WT, the Gfap immunosignal is restricted to the inner third of radial processes. No obvious signal is detected at the inner nuclear layer. The *mdka^mi5001^* upregulates Gfap, and signals are seen at the cell body of Müller glia in the inner nuclear layer. ***B***, Single optical planes from *z*-stack series of the Tg(gfap: EGFP) reporter flat-mount retinal preparation in the *mdka^mi5001^* background. In the ganglion cell and inner plexiform layers, some Müller glia show signs of hypertrophy, including increased levels of the EGFP transgene signal (arrows). ***C***, Cross section view of 3D reconstructed image in the Tg(*gfap:GFP*); *mdka^mi5001^* (green) retina at 28 dpl, immunolabeled with Zpr1 (red) in a flat-mount preparation. Yellow arrows indicate displaced Müller glia somata in the outer plexiform layer. ***D***, Cross section and flat-mounted views of the 3D reconstructed image. Displaced Müller glia (magenta asterisk) retain basal radial process (magenta arrows). opl, Outer plexiform layer; inl, inner nuclear layer; ipl, inner plexiform layer; gcl, ganglion cell layer. Scale bars: ***A***, 30 μm; ***B***, 20 μm.

In all vertebrate retinas, neuronal death induces a gliotic response in Müller glia (Bringmann et al., 2006, 2009; Jadhav et al., 2009). Although the initial reactive gliosis is neuroprotective, persistent gliosis results in dysregulation of retinal homeostasis, glial remodeling and scar formation, and the subsequent death of neurons (Bringmann et al., 2006). In zebrafish, the gliotic response of Müller glia is transient and interrupted by cell cycle entry (Thomas et al., 2016). To determine whether the failure of Müller glia proliferation in Midkine-a mutants leads to a mammalian-like gliotic response, the expression of GFAP was compared in the WT and *mdka^mi5001^* retinas. In unlesioned retinas, immunostaining for GFAP labels the basal processes of Müller glia (Bernardos and Raymond, 2006). In WT retinas at 28 dpl, GFAP immunolabeling resembles that in unlesioned retinas (Fig. 5*A*). In contrast, in the *mdka^mi5001^* retinas at 28 dpl, GFAP immunolabeling is present throughout the cytoplasm, extending apically into the inner nuclear layer (Fig. 5*A*). Enhanced expression of GFAP is a marker of gliosis in mammalian Müller glia (Bringmann et al., 2006). To further characterize the gliotic response in the mutant retinas, we again used the transgenic reporter *Tg(gfap:eGFP)^mi2002^*. Computing the planimetric density of Müller glia in flat-mount preparations at 28 dpl showed no significant difference in the number of Müller glia in wildtype and mutant retinas, suggesting that Müller glia do not die. However, in the *mdka^mi5001^; Tg(gfap:eGFP)^mi2002^* line, Müller glia remained hypertrophic, as evidenced by elevated e*GFP* levels (Fig. 5*B*, arrows; Movies 1, 2), and these cells adopt abnormal morphologies, including expanded lateral extensions in the inner plexiform layer and migration of the somata into the outer plexiform layer (Fig. 5*C*,*D*; Movies 3, 4). This hypertrophic morphology is also revealed in flat mounts stained with the cell junction marker, ZO1, in which the apical profiles of Müller glia have expanded to fill the planar surface of the outer limiting membrane previously occupied by cones (Fig. 4*C*, dashed line). The abnormal gliotic remodeling observed in the mutant retinas is a hallmark of persistent reactive gliosis in mammals.

Movie 1. Confocal *z*-stack series of flat-mount retina in *Tg(gfap:eGFP)^mi2002^*. Müller glia in the WT retina at 28 dpl. 10.1523/JNEUROSCI.1675-19.2019.video.1

Movie 2. Confocal *z*-stack series of flat-mount retina in *mdka^mi5001;^ Tg(gfap:eGFP)^mi2002^* mutant. Müller glia in the *mdka^mi5001^* remain hypertrophic and increased level of the transgene, gfap: EGFP at 28 dpl. 10.1523/JNEUROSCI.1675-19.2019.video.2

Movie 3. 3D reconstruction of the Müller glia in the *mdka^mi5001^* mutant undergo gliotic remodeling. Müller glial somata migrate into the outer plexiform layer at 28 dpl. 10.1523/JNEUROSCI.1675-19.2019.video.3

Movie 4. 3D reconstruction of the Müller glia in the *mdka^mi5001^* mutant undergo gliotic remodeling. The abnormal gliotic remodeling Müller glia in the *mdka^mi5001^* at 28 dpl. 10.1523/JNEUROSCI.1675-19.2019.video.4

### Müller glia in the *mdka^mi5001^* dedifferentiate in response to photoreceptor death

**Figure 6.**
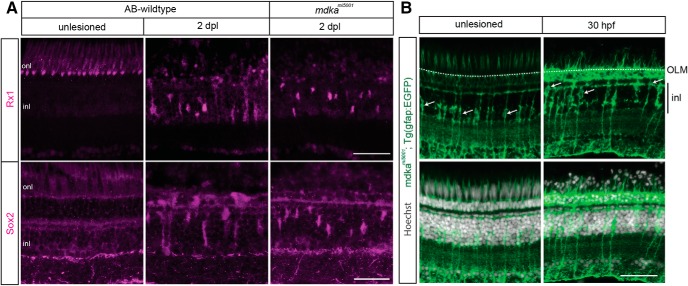
Müller glia in the *mdka^mi5001^* mutant dedifferentiate following photoreceptor death. ***A***, Immunocytochemistry for regeneration-associated genes, Rx1 and Sox2, following photolytic lesion in WT and *mdka^mi5001^* retinas. Lesion induces Rx1 and Sox2 expression in Müller glia both in WT and *mdka^mi5001^* retinas at 2 dpl. ***B***, The *mdka^mi5001^* retinas carrying the Müller glial reporter, *Tg(gfap:eGFP)^mi2002^*. Photoreceptor injury induces interkinetic nuclear migration of Müller glial nuclei in the *mdka^mi5001^* mutant. Arrows indicate cell bodies of Müller glia. The nuclei were stained with Hoechst (gray). OLM, Outer limiting membrane; inl, inner nuclear layer; onl, outer nuclear layer. Scale bars, 30 μm.

**Figure 7.**
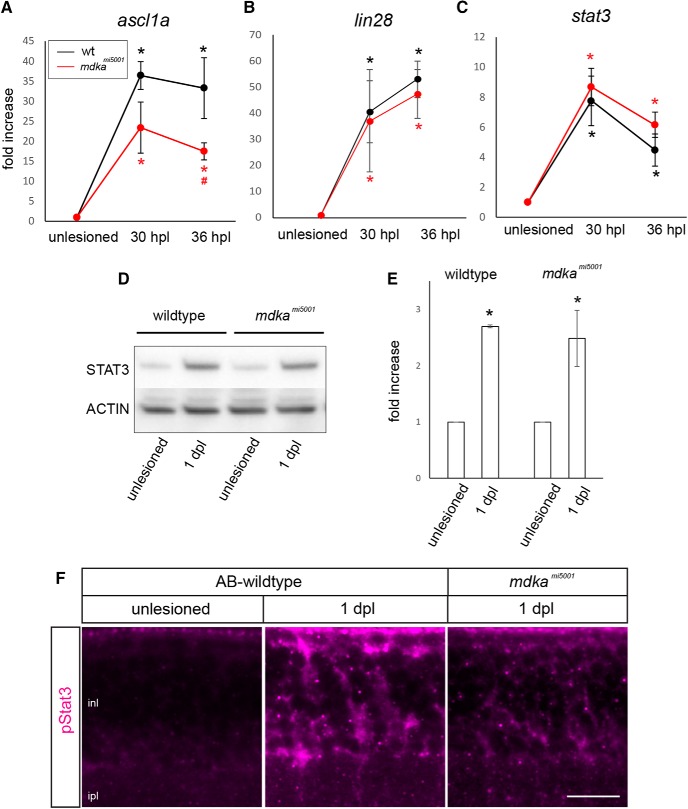
The *mdka^mi5001^* mutant upregulates “core” transcriptional regulators following photoreceptor death. ***A–C***, qPCR for dedifferentiation markers, *ascl1a*, *stat3*, and *lin28*, at 30 and 36 hpl. Both WT and *mdka^mi5001^* upregulate *ascl1a* (***A***; WT: 30 hpl, *p* < 0.0001, 36 hpl, *p* < 0.0001; *mdka^mi5001^*: 30 hpl, *p* = 0.0004, 36 hpl, *p* = 0.006, *p* = 0.0066 relative to WT; F-ratio = 37.5606), *lin28* (***B***; WT: 30 hpl, *p* < 0.0001, 36 hpl, *p* = 0.0095; *mdka^mi5001^*: 30 hpl, *p* < 0.0001, 36 hpl, *p* = 0.0004; F-ratio = 32.9337), and *stat3* (***C***; WT: 30 hpl, *p* = 0.0048, 36 hpl, *p* = 0.0016; *mdka^mi5001^*: 30 hpl, *p* = 0.0004, 36 hpl, *p* = 0.0016), following lesion. ANOVA with post hoc Tukey, relative to unlesioned. ***D***, ***E***, Western blot for Stat3 in WT and *mdka^mi5001^* retinas at 1 dpl. WT: *p* = 0.0002, *mdka^mi5001^*: *p* = 0.0004, ANOVA with post hoc Tukey; F-ratio = 40.8763. ***F***, Immunocytochemistry for phosphorylated Stat3 (pStat3) in the WT and *mdka^mi5001^* mutant. In unlesioned retina, immunosignal for pStat3 is not detected in the inner nuclear layer. Following photoreceptor lesion, Müller glia in the inner nuclear layer upregulate pStat3 in WT, whereas *mdka^mi5001^* mutants have reduced phosphorylation of Stat3. Scale bar, 30 μm. inl, Inner nuclear layer; ipl, inner plexiform layer. *p < 0.01.

In response to neuronal cell death, Müller glia spontaneously reprogram, upregulating stem cell-associated genes before entering the cell cycle (Goldman, 2014; Gorsuch and Hyde, 2014; Lenkowski and Raymond, 2014; Hamon et al., 2016). Immunostaining retinas at 1 and 2 dpl for the stem-cell associated proteins, Rx1 and Sox2, labeled elongated, polygonal nuclei, characteristic of Müller glia (Nagashima et al., 2013; Gorsuch et al., 2017), in both WT and mutant retinas (Fig. 6*A*). Further, the Rx1^+^ and Sox2^+^ nuclei were displaced apically in both (Fig. 6*A*), revealing the interkinetic nuclear migration that is associated with cell cycle progression in Müller glia (Fig. 6*B*) (Nagashima et al., 2013). We also evaluated the reprogramming in Müller glia by qPCR for the core transcriptional factors, *ascl1a*, *stat3*, and *lin28* (Fausett and Goldman, 2006; Ramachandran et al., 2010; Nelson et al., 2012). At 30 and 36 hpl, which is before when Müller glia divide, *ascl1a*, *stat3*, and *lin28* are significantly upregulated in both WT and mutant retinas, although the expression level of *ascl1a* is slightly reduced in the mutants (Fig. 7*B*,*C*). These data indicate that, in the absence of Midkine-a, Müller glia respond to photoreceptor death by reprogramming into a stem cell-like state.

### Midkine-a is partially responsible for *ascl1a* expression via phosphorylation of Stat3

Following a retinal lesion, phosphorylation of Stat3 is required for the upregulation of *ascl1a* in Müller glia (Nelson et al., 2012; Zhao et al., 2014). In WT and *mdka^mi5001^* retinas at 1 and 2 dpl, STAT3 protein was induced (Fig. 7*D*,*E*). Consistent with previously published data (Zhao et al., 2014), at 1 and 2 dpl, pStat3 antibody labels Müller glia in WT animals (Fig. 7*F*). In contrast, in *mdka^mi5001^* retinas at 1 and 2 dpl, pStat3 immunostaining was markedly diminished (Fig. 7*E*), demonstrating that, in Müller glia, Midkine-a regulates the phosphorylation of Stat3, which can explain the reduced expression of *ascl1a* in the mutant retinas.

### Absence of cell cycle progression in mutant Müller glia

**Figure 8.**
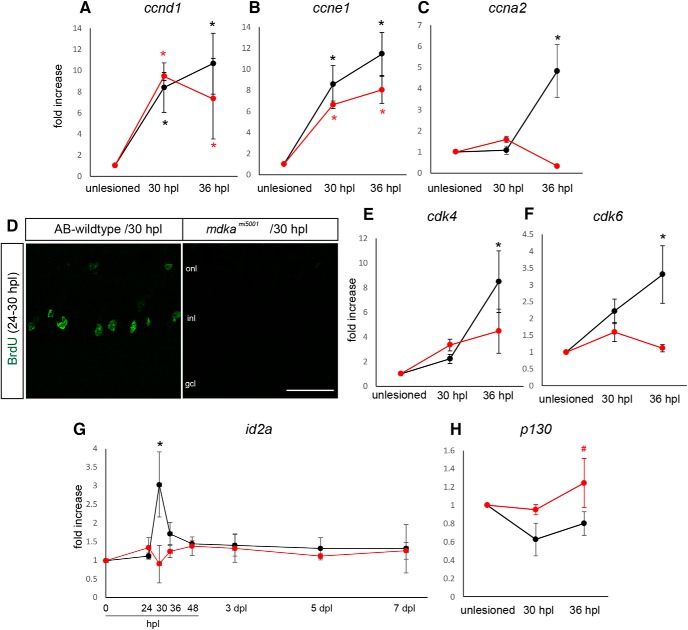
Müller glia in the *mdka^mi5001^* mutant arrest in the G_1_ phase of the cell cycle. ***A–C***, qPCR assay for cell cycle regulator cyclins, *ccnd1*, *ccne1*, and *ccna2* following photolytic lesion. Both WT and *mdka^mi5001^* upregulate G_1_ cyclins, *ccnd1* (***A***; WT: 30 hpl, *p* = 0.0107, 36 hpl, *p* = 0.0013; *mdka^mi5001^*: 30 hpl, *p* = 0.0048, 36 hpl, *p* = 0.0213; F-ratio = 12.0576) and *ccne1* (***B***; WT: 30 hpl, *p* < 0.0001, 36 hpl, *p* < 0.0001; *mdka^mi5001^*: 30 hpl, *p* = 0.0012, 36 hpl, *p* = 0.0001; F-ratio = 36.5808), following lesion (***A***,***B***). The *mdka^mi5001^* mutant fails to upregulate S phase cyclin, *ccna2* (***C***; WT: 36 hpl, *p* = 0.0011; F- ratio = 12.5433). ***D***, S phase assay with BrdU labeling (green) between 24 and 30 hpl. Müller glia in the *mdka^mi5001^* mutants did not incorporate BrdU following lesion. ***E–H***, qPCR of additional cell cycle regulators. Expression of cyclin-dependent kinases, *cdk4* (***E***; WT: 36 hpl, *p* < 0.0001; F-ratio = 15.8783) and *cdk6* (***F***; WT: 36 hpl, *p* = 0.0087; F- ratio = 8.3300), is dysregulated in the *mdka^mi5001^* retinas (***E***,***F***). The WT transiently upregulates *id2a* at 30 hpl (*p* = 0.0003; F- ratio = 7.2578), whereas expression levels did not change in the *mdka^mi5001^* (***G***). Expression of the cell cycle inhibitor, *p130*, decreases in the WT after lesion, whereas *mdka^mi5001^* maintains steady levels of expression (***H***; *p* = 0.002, relative to WT; F- ratio = 6.3823). ANOVA with post hoc Tukey, relative to unlesioned. Scale bar: ***D***, 30 μm. onl, Outer nuclear layer; inl, inner nuclear layer; gcl, ganglion cell layer. *p < 0.03, ^#^*p* < 0.01.

Following reprogramming, Müller glia begin entering the cell cycle around 24 hpl and complete the asymmetric cell divisions by 42 hpl (Nagashima et al., 2013). We next asked whether Müller glia in the *mdka^mi5001^* possess the ability to enter the cell cycle by quantifying the expression of G_1_ phase cyclins, *cyclin d1*(*ccnd1*) and *cyclin e1* (*ccne1*). These cyclins are expressed during G_1_ and function to drive G_1_-to-S phase transition (Dyer and Cepko, 2001). We isolated mRNA at 30 and 36 hpl, knowing that cell cycle progression is not completely synchronous among the population of Müller glia, but that these time points will allow us to capture gene expression changes in Müller glia and exclude Müller glia-derived progenitors. This analysis showed that *mdka^mi5001^* upregulates *ccnd1* and *ccne1* significantly at both 30 and 36 hpl (Fig. 8*A*,*B*), indicating that, following photoreceptor death in the *mdka^mi5001^* mutants, Müller glia enter the G_1_ phase of the cell cycle. In WT retinas, cell cycle entry is followed by upregulation of S phase cyclin, *ccna2* (Fig. 8*C*). In contrast, there is no upregulation of *ccna2* in the *mdka^mi5001^* retinas, indicating that Müller glia in mutants fail to progress from G_1_ to S (Fig. 8*C*). This was confirmed using the S-phase label, BrdU, between 24 and 30 hpl. In WT retinas, Müller glia are uniformly labeled with BrdU; whereas in the in the *mdka^mi5001^* retinas, there are no BrdU-labeled cells (Fig. 8*D*, *n* = 6 retinas). Consistent with these results, the expression of the cell cycle regulators, *cyclin-dependent kinase 4* and *6*, is dysregulated in mutant retinas (Fig. 8*E*,*F*). Together, these results indicate that, following photoreceptor cell death in the *mdka^mi5001^* retinas, cell cycle progression of Müller glia is compromised, demonstrating that, in reprogrammed Müller glia, Midkine-a regulates the G_1_-S phase transition.

During retinal development, Midkine-a governs cell cycle kinetics through Id2a (Luo et al., 2012). Id proteins play important roles in cell cycle regulation during development and in cancer (Lasorella et al., 1996; Sikder et al., 2003). In WT retinas, *id2a* expression is markedly upregulated at 30 hpl, as Müller glia progress through the cell cycle, and rapidly returns to baseline levels by 48 hpl, when the single asymmetric mitotic division is complete (Fig. 8*G*). This transient induction of *id2a* is completely absent in the *mdka^mi5001^* retinas (Fig. 8*G*). In cancer cells, Id2 proteins antagonize the Rb family of cell cycle inhibitors, thereby allowing progression from G_1_ to S phase of the cell cycle (Lasorella et al., 2001; Sikder et al., 2003). Previous analyses of the Müller glia specific transcriptome show that *p130*, one of the Rb gene family, exhibits highest expression among Rb genes in quiescent Müller glia (Sifuentes et al., 2016; Nieto-Arellano and Sánchez-Iranzo, 2019). Consistent with these data, we validated that, in WT retinas, the expression of p130 decreases as Müller glia progress through the cell cycle (Fig. 8*H*). In contrast, in *mdka^mi5001^* retinas at 30 and 36 hpl, p130 levels are elevated above the those found in quiescent Müller glia (Fig. 8*H*). These results suggest that Id2a is downstream of Midkine-a, and in Müller glia Id2a functions to inhibit Rb genes.

### Signaling through the ALK receptor is responsible for Müller glial proliferation

**Figure 9.**
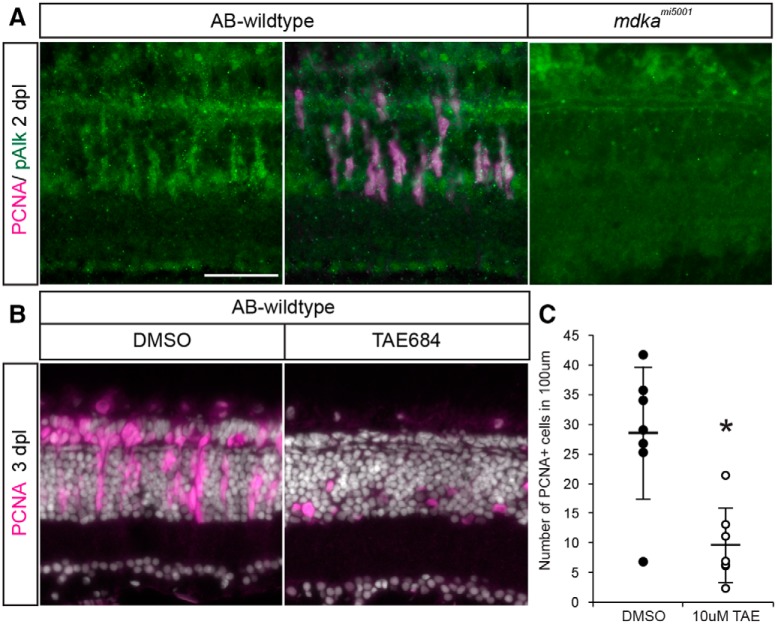
Activation of the ALK receptor is required for Müller glial to proliferate. ***A***, Double immunostaining for PCNA (magenta) and pAlk (green). PCNA^+^ cells express pAlk in WT retina at 2 dpl, whereas pAlk immunostaining was not detected in *mdka^mi5001^* retina. Scale bar, 30 μm. ***B***, Pharmacological inhibition of Alk using TAE684 suppresses proliferation in the WT retinas after a lesion. ***C***, Counts of PCNA^+^ proliferative cells in DMSO- or TAE684-treated WT retinas at 3 dpl. *n* = 7. *p = 0.0011 (nonparametric Mann-Whitney-Wilcoxon).

ALK is a member of the superfamily of receptor tyrosine kinases. ALK is involved in the initiation and progression of many cancers, including neuroblastoma (Morris et al., 1995; Webb et al., 2009; Hallberg and Palmer, 2013). Midkine and its related protein pleiotrophin are the only ligands known to activate ALK (Stoica et al., 2001, 2002). To determine whether Alk functions as a Midkine-a receptor on Müller glia during photoreceptor regeneration, double immunocytochemistry was performed for pAlk and PCNA following a photolytic lesion. In WT retinas, pAlk colocalizes with PCNA, indicating activation of Alk in dividing Müller glia and Müller glia-derived progenitors (Fig. 9*A*). In contrast, both pAlk and PCNA immunolabeling were absent in *mdka^mi5001^* retinas, indicating that, in the retina, Midkine-a is required for ALK phosphorylation. To test whether activation of ALK is required for proliferation among Müller glia, WT animals were housed from 24 to 72 hpl in the ALK inhibitor, TAE684. Inhibiting the activation of ALK phenocopied the proliferation defect observed in the *mdka^mi5001^* mutants (Fig. 9*B*,*C*). These data indicate that phosphorylation of Alk is required for Müller glia to proliferate and identify ALK as a putative receptor for Midkine-a during the initial asymmetric division in Müller glia and the subsequent regeneration of cone photoreceptors.

## Discussion

In mammals, neuronal damage in the retina stimulates transient entry of Müller glia into the cell cycle; however, any subsequent proliferative response is very limited (Dyer and Cepko, 2000; Hamon et al., 2019; Rueda et al., 2019). In zebrafish, Müller glia respond to neuronal death by spontaneously entering and transiting the cell cycle, giving rise to Müller glia-derived progenitors that amplify in number and functionally replace ablated neurons. Numerous studies have identified transcriptional regulators and signaling cascades that promote Müller glia reprogramming in both mammals and fish (Karl and Reh, 2010; Goldman, 2014; Gorsuch and Hyde, 2014; Lenkowski and Raymond, 2014; Hamon et al., 2016). The molecular mechanisms that govern cell cycle kinetics in Müller glia, while essential, have received relatively little attention. Here we provide the first evidence that Midkine-a, ostensibly acting as an extrinsic regulator of proliferation, governs the transition from G_1_ to S phases of the cell cycle in injury-induced, reprogrammed Müller glia.

**Figure 10.**
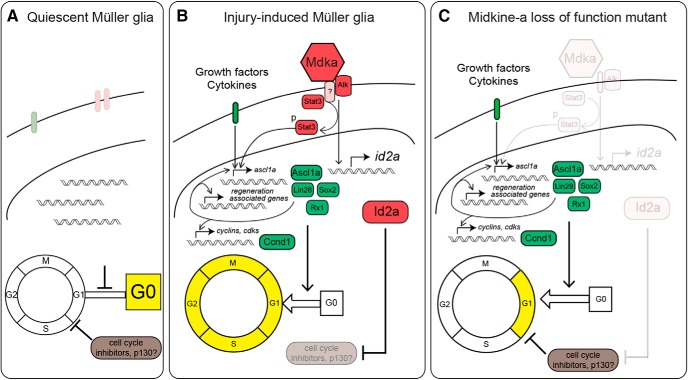
Model of Midkine-a-mediated cell cycle regulation in Müller glia. ***A***, In quiescent Müller glia, cell cycle inhibitors keep cells in G_0_ phase. ***B***, Injury induces cytokines and growth factors to upregulate regeneration-associated reprogramming genes for dedifferentiation and cell cycle reentry. Midkine-a-Alk signaling participates in the induction of the regeneration-associated gene, *ascl1a*, via phosphorylation of Stat3. Midkine-a signaling also induces expression of the cell cycle regulator, *id2a*, that inhibits cell cycle inhibitors, such as p130. ***C***, In the absence of Midkine-a, Müller glia fail to suppress cell cycle inhibition, resulting in compromised progression of the cell cycle.

Our data support the mechanistic model shown in Figure 10. In unlesioned retinas, Müller glia remain quiescent in the G_0_ phase (Fig. 10*A*). In response to photoreceptor cell death, nearby Müller glia upregulate reprogramming-associated genes, and enter the cell cycle (Fig. 10*B*). Midkine-a, signaling through Alk receptors, promotes the expression of *ascl1a* via phosphorylation of Stat3 (Fig. 10*B*). Midkine-a also induces the brief, transient upregulation of Id2a, which suppresses cell cycle inhibitor p130, thereby allowing Müller glia to enter both S and the subsequent phases of the cell cycle (Fig. 10*B*). In Midkine-a loss-of-function mutants, Müller glia initiate reprogramming into a stem cell state and enter G_1_ phase of the cell cycle, but fail to activate Id2a and fail to transition from G_1_ to S and mitosis (Fig. 10*B*,*C*). The consequence of this cell cycle block is the selective failure in the regeneration of cone photoreceptors.

During retinal development, Müller glia emerge from late-stage retinal progenitors, and cell cycle inhibitors play pivotal roles in their fate determination (Turner and Cepko, 1987; Furukawa et al., 2000; Levine et al., 2000; Ueki et al., 2012; Del Debbio et al., 2016). In adult retinas, Müller glia retain an intrinsic genetic program that is shared with these retinal progenitors (Roesch et al., 2008). Müller glia in mammalian retinas respond to neuronal death by entry into the cell cycle (Joly et al., 2011; Nomura-Komoike et al., 2016). Importantly, in the mouse, genetic modifications that allow Müller glia to persistently express *cyclin* genes are sufficient to promote their mitotic division (Hamon et al., 2019; Rueda et al., 2019). Our data, together with these observations, suggest that Müller glia in both mammals and zebrafish possess similar mechanisms that function to integrate injury-related signals from dying neurons and promote entry into the cell cycle, but in zebrafish, Midkine-a serves as a unique extrinsic signal that allows these cells to proliferate.

Following photoreceptor death, reprogrammed Müller glia enter the cell cycle within 24 hpl and undergo a single division by 42 hpl (Nagashima et al., 2013). This temporal sequence is closely coordinated, but not completely synchronized. We timed experiments using RNA isolated from whole retinas such that cell cycle-related gene expression could be evaluated in Müller glial stem cells while excluding Müller glia-derived progenitors. Following photoreceptor death in zebrafish, *id2a* is upregulated transiently at 30 hpl and returns to baseline levels by 36 hpl. This indicates that Midkine-a-dependent Id2a expression is required for the proliferation of Müller glia, but not Müller glia-derived progenitors. The family of Id proteins is involved in intrinsic control of proliferation during development and in cancer (Ruzinova and Benezra, 2003; Sikder et al., 2003). Id2 regulates the Rb proteins, and high levels of Id2 can suppress the Rb tumor suppressor pathway, which blocks progression from G_1_ to S phase of the cell cycle (Lasorella et al., 2000, 2001). In adult mice, the Rb-family member p130 maintains quiescence in muscle satellite cells, which retain the capacity to self-renew and regenerate myoblasts (Carnac et al., 2000). During sensory hair cell regeneration in zebrafish inner ear, p130 is downregulated immediately following injury (Jiang et al., 2014). Consistent with these data, our *in silico* screening identified p130 as a highly expressed gene in quiescent Müller glia, suggesting that p130 expressed in Müller glia of uninjured retinas functions to restrict their proliferation (Sifuentes et al., 2016; Nieto-Arellano and Sánchez-Iranzo, 2019). Our data suggest that, in response to cell death, Midkine-a signaling blocks p130 through upregulating Id2a, allowing Müller glia to progress through the cell cycle. We suggest that the brief upregulation and downregulation of *id2a* are a mechanism that allows Müller glia to divide, but restricts these cells to a single mitotic cycle. Further, our data suggest that Midkine-a is required for the rising phase of this transient *id2a* expression. Notably, increased levels of Id2 are also present in anaplastic large cell lymphomas that result from constitutive activation of the Midkine receptor, ALK (Mathas et al., 2009). It is not known whether ALK in Müller glia functions alone or as a member of multiprotein complex to relay the Midkine-a signal. Receptor protein tyrosine phosphatase-ζ (RPTP-z) is also a known receptor for Midkine and can activate the intracellular kinase domain of ALK, and may function as a coreceptor to transduce Midkine-a signaling in Müller glia (Mathas et al., 2009; Hallberg and Palmer, 2013).

Following photoreceptor death in the *mdka^mi5001^* mutants, Müller glia initially fail to progress through the cell cycle, although a small number of Müller glia eventually do so. As a consequence, the regeneration of cone photoreceptors is permanently compromised, whereas the regeneration of rod photoreceptors is not. This suggests the initial proliferative response of the Müller glia gives rise to cone progenitors. Previous reports suggest that separate lineages give rise to regenerated cone and rod photoreceptors, respectively (Morris et al., 2008; Thummel et al., 2010; Gorsuch et al., 2017), and our results are consistent with these reports. We favor the interpretation that fate-restricted rod precursors persist in the outer nuclear layer and contribute to the regeneration of rod photoreceptors. However, we cannot exclude the possibility that regenerated rods in the *mdka^mi5001^* mutants originate from the few latent Müller glia that progress through the cell cycle.

Heterogeneity of stem and progenitor populations is a clinical challenge when treating malignant tumors, where Midkine is highly expressed (Muramatsu, 2011; Sakamoto and Kadomatsu, 2012). Müller glia share common features of quiescence, self-renewal, and multipotency with cancer stem cells. In unlesioned retina, Müller glia are quiescent and sporadically divide and produce fate-restricted rod precursor (Raymond and Rivlin, 1987; Stenkamp, 2011). Cell death reprograms Müller glia to a stem cell-like state (Karl and Reh, 2010; Goldman, 2014; Gorsuch and Hyde, 2014; Lenkowski and Raymond, 2014; Hamon et al., 2016). *In vitro* experiments demonstrated that inhibition of Midkine successfully suppresses proliferation of cancer stem cells (Mirkin et al., 2005; Erdogan et al., 2017). Therefore, Midkine silencing is proposed as a potential therapy for limiting cell cycle progression in cancer stem cells (Muramatsu and Kadomatsu, 2014). Our data also provide molecular insights into the potential role of Midkine in tumorigenesis, especially in regulating the cell cycle among cancer stem cells.

Constitutive activation of glial cells and formation of a glial scar are detrimental to the function of the CNS. An intriguing phenotype in the *mdka^mi5001^* mutants is the cell death-induced gliotic remodeling of Müller glia. A previous report showed that pharmacological suppression of cell cycle progression following photoreceptor death results in hypertrophy and increased GFAP in Müller glia (Thomas et al., 2016). Together, these results suggest that, in zebrafish Müller glia, the molecular mechanisms that promote cell cycle progression are required to limit the initial gliotic response. Although it is not clear whether entry into the cell cycle initiates reactive gliosis in mammalian retinas, levels of cell cycle proteins appear to be a critical variable in the gliotic response (Dyer and Cepko, 2000; Levine et al., 2000; Vázquez-Chona et al., 2011; Ueki et al., 2012).

Our data significantly expand the understanding of retinal regeneration in zebrafish and more fully define the function of Midkine-a in governing the eukaryotic cell cycle. We provide convincing evidence that Midkine-a regulates proliferation of reprogrammed Müller glial during the regeneration of cone photoreceptors. In the absence of Midkine-a, zebrafish Müller glia respond similarly to Müller glia in mammals, with only a limited ability to regenerate neurons. In developing mammalian retinas, Midkine has been identified as component in the core transcriptional repertoire of retinal progenitors (Livesey et al., 2004). It remains to be determined whether Midkine-dependent cell cycle machinery is present in the Müller glia of adult mammals or whether manipulation of Midkine signaling in adult mammals could promote neuronal regeneration.
